# Preliminary study on an added vestibular-ocular reflex visual conflict task for postural control

**Published:** 2020-04-16

**Authors:** Ryan N. Moran, Graham Cochrane

**Affiliations:** ^1^Department of Health Science, The University of Alabama, Tuscaloosa Alabama, United States; ^2^Department of Physical Therapy, University of Alabama at Birmingham, Birmingham, Alabama, United States

**Keywords:** balance, vestibular, visual

## Abstract

**Background:**

Using the modified-Clinical Test of Sensory Integration and Balance (m-CTSIB), clinicians can assess sensory feedback systems of the visual, vestibular, and somatosensory systems on postural control. However, with growing vestibulo-ocular reflex (VOR) assessment, the addition of a VOR task, for sensory feedback on postural control has yet to be investigated.

**Aim:**

The aim of the study was to examine the preliminary effect of an added VOR visual conflict task during postural control conditions of the m-CTSIB at baseline and re-test reliability.

**Methods:**

Seventeen healthy college-aged individuals completed a baseline m-CTSIB with an added VOR visual conflict condition consisting of a lateral headshake and follow-up assessment occurring 72-h after baseline. Measures consisted of m-CTSIB sway scores on individual conditions of eyes open and eyes closed tasks on firm and foam surfaces. A series of Wilcoxon matched-pairs signed-rank tests were conducted to determine the differences between the VOR condition and the m-CTSIB conditions. A Spearman Rank Order correlation was used to examine the retest reliability.

**Results:**

The VOR visual conflict task condition produced worse sway index scores than eyes-open firm and foam (p<0.001), but better scores than eyes-closed foam (*P*=0.01) conditions at baseline. VOR tasks on their respective firm (r_s_=0.81) and foam surface (r_s_=0.83) were strongly correlated at 72-h retest.

**Conclusions:**

The addition of a VOR visual conflict task condition differed from the other conditions of the m-CTSIB, further targeting the vestibular-ocular system from the vestibular-spinal system during postural control. Incorporating a VOR task during postural stability may create greater postural control deficits in individuals with vestibular-ocular dysfunction. Test-retest correlations at 72-h were clinically acceptable.

**Relevance for patients:**

Addition of a VOR task as visual conflict condition of the m-CTSIB may assist in additional sensory system feedback for concussion assessment.

## 1. Introduction

Consensus statements for concussion recommend medical assessment of balance and gait, along with vestibular and ocular function [1]. While the Balance Error Scoring System (BESS), a human-error scored balance assessment is the recommended balance assessment on the Sport Concussion Assessment Tool (SCAT) [2], more objective tools have been considered in the literature, including the Sensory Organization Test (SOT) and the Clinical Test of Sensory Integration and Balance (CTSIB), to provide further insight into balance and postural control [3]. These assessments have gained interest, due to their ability to used advanced, laboratory technology to quantify various pathways of postural control.

One of the most commonly used laboratory tools is the SOT, which uses a force platform and a harness cage to disrupt visual and somatosensory senses by reducing spatial awareness through the moving cage and platform [4]. However, the SOT has been criticized for its ability to detect subtle balance and vestibular deficits [5-7]. In an attempt to improve the delineation of postural control performance, a headshake task was added to the SOT (Head-Shake Sensory Organization Test [HS-SOT]) [8-11]. The previous research has found that the addition of a horizontal head-shake task during the SOT decreased postural control [12] and was able to identify unilateral vestibular dysfunction [11] and asymmetry [9]. In addition, Honaker *et al*. [9] recommend implementing a 15°/s headshake task to the SOT for vestibular screening. However, the HS-SOT is completed with an eyes closed task, which may not accurately represent contribution of the vestibular-ocular pathway to postural stability, due to the need to have the eyes open to maintain visual stability during head movement [11,13]. In addition, with a moving platform and cage, paired with the computer posturography and cost of the SOT , this test may not be the best to apply laboratory results clinically [3].

The CTSIB utilizes similar conflicting sensory input assessment as the SOT, to assess postural control. More recently, a modified version of the CTSIB (m-CTSIB) has been developed to use force-platform technology, in a static environment (i.e., non-moving platform), with platform (firm), and foam surfaces [4]. The m-CTSIB is unique in that it evaluates the three systems of the body (i.e., visual, vestibular, and somatosensory) that maintain postural control, without the use of computerized posturography [14,15]. However, the vestibular system is difficult to isolate and is comprised various neurophysiological pathways, such as the somatosensory system, vestibular-spinal tract, and the vestibular-ocular tract [16]. Specifically, the vestibular-spinal tract’s primary function is to maintain balance [16,17], whereas the vestibular-ocular system and reflex (VOR) functions to maintain visual stability during head movements [16]. Since the vestibular-spinal and vestibular-ocular pathways do not share identical neuronal circuitry [18], it is important to attempt to decipher their respective evaluation related to vestibular assessment. While sharing separate brain circuitry, these pathways are interdependent; gaze stabilization is impaired on an unstable body, and stable gaze is important for proper postural control [19]. In addition, previous SOT research has utilized the headshake during eyes-closed tasks, whereas an eyes-open task will provide visual stability on the vestibular-ocular system, not accounted for during the HS-SOT.

Recently, VOR assessment has received consideration separate from vestibular-spinal assessment in concussion as part of the Vestibular/Ocular Motor Screening (VOMS). The VOMS test examines symptom provocation in vestibular-related symptoms of headache, dizziness, nausea, and fogginess, following individual ocular tasks, including smooth pursuits, saccades, and convergence, and vestibular tasks, including VOR and visual motion sensitivity. However, it remains unclear if VOR assessment and vestibular-spinal assessment share vestibular outcomes when examined together or may jeopardize collective vestibular assessment. It has been suggested that changes to the VOR are associated with changes to vestibular information weighting in postural control tasks [20]; therefore, combining both VOR tasks and postural control tasks may be clinically useful.

Importantly, utilization of the VOMS requires subjective symptom reporting. As it has been estimated that upward of 55% of athletes does not properly report concussions and their symptoms [21-23], assessing VOR alone as a symptom provocation test of the VOMS may jeopardize the validity of VOR assessment. While the SOT and m-CTSIB have been reported to be useful outcome measures for changes in postural control, and strong coefficients have been found on the re-test of the HS-SOT conditions, it is important to establish test-retest reliability of the m-CTSIB and an added headshake task. As the BESS test has been reported to have performance return to baseline 3-5 days post-injury [24], 3 days/72 h test-retest, which has also been used as a common test-retest interval [25,26], may be clinically useful. Test-retest reliability will allow comparison of the visual conflict task of the m-CTSIB stability over time to that of the HS-SOT. Therefore, the purpose of this preliminary study was to examine an added VOR visual conflict task as a separate test condition to examine differences between the normal conditions of the m-CTSIB. It was hypothesized that adding a VOR visual conflict task during postural control assessment may present differently on sway scores compared to the standard eyes-open and eyes-closed tasks. A secondary purpose is to determine the reliability of the VOR visual conflict tasks as a condition on the m-CTSIB.

## 2. Materials and Methods

### 2.1. Participants and procedures

A total of 17 healthy, college-aged individuals (20.7±2.3 years) participated in the study. Of the 17 participants, nine were male and eight were female. All participants were free of any learning disabilities, history of concussion, and any vestibular, visual, or balance disorders through self-report diagnoses. Participants completed a baseline m-CTSIB with allsix conditions, in a quiet research laboratory. Seventy-two hours later, all participants completed a re-test of the m-CTSIB. Before data collection, all participants completed an informed consent and a demographic questionnaire that consisted of age, sex, and pertinent medical information to exclude any participants.

### 2.2. Measures

The modified Clinical Test of Sensory Integration and Balance (m-CTSIB) is a postural control test conducted on the Biodex Balance System (Biodex Medical Systems, Shirley, NY, USA) that used a force-platform to assess the sensory selection process by compromising available visual, vestibular, and somatosensory senses. The m-CTSIB provides an assessment for how well an individual can integrate these three senses and compensate when one or more of those senses are compromised [14,15]. The m-CTSIB consists of four test conditions, assessed for 20-s each, while the participant stands at the center of the force-platform, without shoes and socks on, and with their feet shoulder width apart and hands resting comfortably on their hips. The m-CTSIB consists of the following: (1) Eyes-open firm surface (incorporates visual, vestibular, and somatosensory inputs), (2) eyes-closed firm surface (eliminates visual input to examine vestibular and somatosensory inputs), (3) eyes-open foam surface (compromises somatosensory input to examine visual and vestibular inputs), and (4) eyes-closed foam surface (compromises somatosensory and visual input to examine vestibular input). For the purpose of this study, two additional conditions were added to the m-CTSIB protocol, which consisted of an added VOR visual conflict task on the firm and foam surface. The VOR tasks consisted of the horizontal VOR task from the VOMS [16], with the same participant positioning. The patient is asked to rotate their head horizontally while maintaining focus on the single, stationary target that is on the screen of the Biodex Balance System. The head is moved at an amplitude of 20 degrees to each side and a metronome is used to ensure the speed of rotation which is maintained at 180 beats/min [16]. VOR task-firm surface enables partial visual input, but information conflicts with vestibular information to examine more vestibular and somatosensory inputs. VOR task-foam surface examines the mediation of visual, vestibular, and somatosensory inputs. A sway index score is objectively calculated as the participants standard deviation of the sway angle and direction from center, with lower scores indicating better stability and control.

### 2.3. Statistical analysis

A series of Wilcoxon matched-pairs signed-rank tests were used to examine the differences between the VOR visual conflict condition on its respective surface, either firm or foam, to eyes-open and eyes-closed m-CTSIB condition of that same surface. Due to non-parametric data, Spearman rank order correlations were analyzed to determine the strength of the association between baseline and a 72-h retest interval. The criteria for interpreting the strength of the correlation was weak (0-0.3), moderate (0.3-0.7), or strong (0.7-1.0) [27]. The standard error of the means (SEM) was examined, as well as the minimal detectable change (MDC), which was calculated [28] as 1.96 × SEM × √2. The SEM is defined as the estimate of the variability between the scores obtained and the participants “true” score [29]. The MDC reflects the smallest change in the score that can be interpreted as real and not from measurement error [28].

## 3. Results

At baseline, the VOR visual conflict task condition produced worse sway index scores on the firm surface than eyes-open (*P*<0.001) (Table 1). Differences existed on VOR foam surface and eyes-open (*P*<0.001) and eyes-closed (*P*=0.01). However, on the foam surface, eyes-closed condition, the VOR visual conflict task had better sway index scores than the m-CTSIB condition. No differences were noted between the eyes-closed firm surface and VOR visual conflict firm surface (*P*=0.51).

**Table 1:** m-CTSIB condition performance including VOR visual conflict task.

m-CTSIB condition	Sway index score	Median (IQR)	*P*-value	Effect size
Eyes-open firm	0.43±0.13	0.37 (0.21)	0.001*	0.58

Eyes-closed firm	0.65±0.28	0.61 (0.25)	0.51	1.1

VOR conflict firm	0.66±0.15	0.67 (0.20)	-	

Eyes-open foam	0.68±0.12	0.60 (0.28)	0.000*	0.62

Eyes-closed foam	1.82±0.43	1.96 (0.60)	0.01*	0.40

VOR conflict foam	1.52±0.35	1.34 (0.48)	-	

VOR condition (firm or foam surface) only compared to their respective surface;

* =significant difference between VOR visual conflict of the same surface. VOR: vestibulo-ocular reflex

At 72 h retest, VOR visual conflict task was strongly correlated on firm (r_s_=0.815, *P*<0.001) and foam surface (r_s_=0.830, *P*<0.001; Table 2). Regarding the m-CTSIB conditions, at re-test, there was a poor correlation for the eyes-open firm surface (r_s_=0.144, *P*=0.58), eyes-open foam surface (r_s_=0.146, *P*=0.57), and eyes-closed foam surface (r_s_=0.077, *P*=0.76). However, there was moderate correlation between the eyes-closed firm surface (r_s_=0.606, *P*=0.01). Regarding SEM and MDC, 14 (82%) displayed a change in sway index scores greater than the SEM on VOR conflict firm and 11 (65%) on VOR conflict foam surfaces. A total of 9 (53%) participants displayed a change that exceeded the MDC for VOR conflict firm surface and 3 (17%) on VOR conflict foam surface.

**Table 2:** Reliability of the m-CTSIB and VOR visual conflict conditions.

m-CTSIB condition	r_s_	SEM	MDC
Eyes-open firm	0.144	0.032	0.88

Eyes-closed firm	0.606	0.063	0.17

VOR conflict firm	0.815	0.043	0.11

Eyes-open foam	0.146	0.037	0.10

Eyes-closed foam	0.077	0.078	0.21

VOR conflict foam	0.830	0.100	0.27

m-CTSIB: modified-clinical test of sensory integration and balance, VOR: Vestibulo-ocular reflex, MDC: Minimal detectable change, SEM: Standard error of the means

## 4. Discussion

This is believed to be the first study to examine the differences between an added VOR visual conflict task to the m-CTSIB to provide preliminary understanding to the role of the vestibular-ocular system, in conjunction with the visual, vestibular-spinal, and somatosensory system during postural control. While the previous research on the HS-SOT has failed to specifically target the VOR, similar results have been noted with the current study. The addition of the headshake task on the SOT lead to decreased postural control on both the firm surface and sway-induced/tilted cage conditions [8], which is consistent with the findings of this study on the eyes-open firm and foam conditions. The results of our study finding that the VOR added task had better sway scores than the eyes-closed foam surface condition can be partially supported by improvements in the HS-SOT conditions on the firm condition in healthy controls, with worse scores on the sway-referenced/tilted cage condition with the added headshake [9]. This findings can possibly be explained by the addition of head movements tasks, whether VOR or semicircular canals, creates postural instability or decreasing control, due to changes in head orientation [11,30-33]. By adding a VOR task, the headshake leads to activation of semicircular canals to add an additional sensory integration strain [11,30,34], and more specifically the vestibular-ocular reflex [9,35], forcing the brain to decipher between head movement and movement due to postural sway in determining the proper righting reflex. Therefore, the addition of the VOR task caused a degradation of postural control in all conditions, but the eyes-closed firm surface condition. It may be possible that the reintroduction of vision to this condition led to improvement in postural control despite activation of the semicircular canals. Worse control with an added VOR task may also be due to asymmetrical neural input within the vestibular nuclei after stimulation of the vestibular structures (i.e., semicircular canals and VOR), changing asymmetry within the velocity storage integrator [9,11,34,36].

While the m-CTSIB uses force-plate technology, other balance and postural control assessment tools such as the modified Balance Error Scoring System (m-BESS), which is used as the consensus sideline balance assessment for concussion [2], does not require equipment, nor a foam surface. If applying the results of the current study to the m-BESS eyes-closed, double leg stance on a firm surface, it appears that an additive VOR visual conflict may not be able to distinguish any VOR deficits, as no differences were noted in the current study between the VOR visual conflict and eyes closed, firm surface. While the m-BESS uses an error scoring system to score performance, overall sway index should not differ extensively.

This is believed to be the first study to attempt to examine the reliability of the VOR visual conflict task as a condition on the m-CTSIB. Our results are similar to the non-modified CTSIB which was found to have an interclass correlation coefficient of 0.98 in older adults [37]. Results are also similar to those of the HS-SOT, reporting coefficients of 0.85 (firm surface) and 0.78 (sway-referenced/tilted cage surface) on the two headshake conditions in youth and 0.64 and 0.55, respectively, in adults [10]. With good reliability of the m-CTSIB with a headshake, this may provide early value to the m-CTSIB to assess changes and sensory input in postural control approximately at 3-days’ time. At re-test, VOR task scores were 0.75-1.59 on firm and foam, respectively, which is a slight increase in postural sway scores, which may also provide early insight into a lack of a learning or practice effect during m-CTSIB and VOR added tasks over time. As the standard m-CTSIB conditions had poor to moderate reliability at 72-h re-test, the greater stability of the VOR visual conflict conditions may have increased utility as performance is less likely to vary. We hypothesize that the low reliability for the m-CTSIB conditions may be low compared to the VOR visual task due to the use of multiple systems competing at one time to provide postural control, whereas the VOR task may assist postural control by focusing on one pathway, which is the VOR, a shared vestibular pathway as the vestibular-spinal pathway.

This study is not without limitations. First, the study was completed on a pilot sample of healthy college-aged individuals, so results may vary in different populations, including ages and in athletes. In addition, vestibular symptoms were not asked before or post-condition testing, similar to the VOMS assessment, to understand if vestibular-ocular related symptoms (i.e., dizziness, and fogginess) were factors or predictors for VOR visual conflict performance for postural control. While overall sway index scores provide an accurate understanding of postural control, as clinicians will examine concussed individuals with gross instability and swaying, center of pressure coordinates may shed additional information on specific directions of sway during VOR tasks. It may be beneficial for future research to address the use of a VOR (headshake) task on the m-CTSIB longitudinally post-concussion, but also as a predictor or outcome measure compared to other clinical measures for individuals with vestibular deficits or vestibular-ocular dysfunction both at baseline and post-concussion. Furthermore, using headshake velocity (i.e., 60°/s) at different speeds may present with different results, as Mishra *et al*. noted varying velocities may increase the sensitivity for identifying patients with unilateral peripheral vestibular deficits [11]. The previous research by Honaker *et al*. [34] includes using a head mounted rate sensor as opposed to a metronome, where reliance on auditory signaling and no distinct quantified measurement of head movement may alter results. Having more quantifiable VOR information may also be able to identify competing systems.

## 5. Conclusions

The addition of a VOR visual conflict task during the m-CTSIB differed between the eyes-open firm surface and both eyes-open and closed foam surface conditions. These results provide early insight into the incorporation of a visual conflict during balance and postural control assessment to provide a better understanding of visual, vestibular-spinal, vestibular-ocular, and somatosensory senses during concussion assessment. Further, the VOR visual conflict task for both surfaces (i.e., firm and foam) was strongly correlated 72-h post-baseline testing, which reflects the consistency between short-term retest intervals. Future research is needed to quantify vestibular-like changes post-concussion and longitudinally to further validate the effectiveness of a VOR visual conflict task for postural control. To the best of our knowledge, this is the first study to evaluate the VOR visual conflict task on the m-CTSIB and its reliability over time.

### Disclosures

### Funding

No funding was obtained for this study.

### Conflict of interest statement

The authors declare no conflicts of interest.

### Ethical approvals

The University of Alabama Institutional Review Board reviewed and approved this study.
